# Sexual health and wellbeing after pelvic radiotherapy among women with and without a reported history of sexual abuse: important issues in cancer survivorship care

**DOI:** 10.1007/s00520-021-06263-0

**Published:** 2021-05-18

**Authors:** Linda Åkeflo, Eva Elmerstig, Gail Dunberger, Viktor Skokic, Amanda Arnell, Karin Bergmark

**Affiliations:** 1 Division of Clinical Cancer Epidemiology, Department of Oncology, Institute of Clinical Science, The Sahlgrenska Academy, University of Gothenburg, 413 45 Gothenburg, Sweden; 2 Centre for Sexology and Sexuality Studies, Malmö University, Malmö, Sweden; 3 Department of Health Care Sciences, Ersta Sköndal Bräcke University College, Stockholm, Sweden

**Keywords:** Female cancer survivor, Sexual abuse, Pelvic radiotherapy, Late effect, Sexual health, Sexual dysfunction

## Abstract

**Aims:**

Sexual abuse is a women’s health concern globally. Although experience of sexual abuse and cancer may constitute risk factors for sexual dysfunction and low wellbeing, the effects of sexual abuse have received little attention in oncology care. This study aims to explore sexual health and wellbeing in women after pelvic radiotherapy and to determine the relationship between sexual abuse and sexual dysfunction, and decreased wellbeing.

**Methods:**

Using a study-specific questionnaire, data were collected during 2011–2017 from women with gynaecological, anal, or rectal cancer treated with curative pelvic radiotherapy in a population-based cohort and a referred patient group. Subgroup analyses of data from women with a reported history of sexual abuse were conducted, comparing socio-demographics, diagnosis, aspects of sexual health and wellbeing.

**Results:**

In the total sample of 570 women, 11% reported a history of sexual abuse and among these women the most common diagnosis was cervical cancer. More women with than without a history of sexual abuse reported feeling depressed (19.4% vs. 9%, p = 0.007) or anxious (22.6% vs. 11.8%, p = 0.007) and suffering genital pain during sexual activity (52% vs. 25.1%, p = 0.011, RR 2.07, CI 1.24–3.16). In the total study cohort, genital pain during sexual activity was associated with vaginal shortness (68.5% vs. 31.4% p ≤ 0.001) and inelasticity (66.6% vs. 33.3%, p ≤ 0.001).

**Conclusions:**

Our findings suggest that a history of both sexual abuse and pelvic radiotherapy in women are associated with increased psychological distress and sexual impairment, challenging healthcare professionals to take action to prevent retraumatisation and provide appropriate interventions and support.

## Introduction

Sexual abuse and intimate partner violence are problems faced by women worldwide, often causing life-long physiological and psychological consequences, and sexual dysfunctions such as impaired sexual desire, sexual pain, reduced lubrication and orgasm disorders [1–3]. According to the World Health Organization (WHO), sexual abuse affects up to a third of women in the general population [4–6]. Evidence suggests that sexual abuse increases the risk of some cancer diagnoses associated with human papillomavirus (HPV) infection. HPV is a common sexually transmitted infection (STI) that plays an important role in almost all cervical cancers, a high proportion of anal cancers, and some cancers of the vagina, vulva, and oropharynx [7, 8]. Most women in the world are likely to be infected with one or more types of HPV during their sexual life, which in most cases clears but in some rare cases causes a persistent HPV infection that can develop into potential premalignant dysplasia and cancer [9]. One of several coping strategies for dealing with the trauma of sexual abuse is avoidance of regular cervical screening, risking diagnosis of cancer at a later stage. A previous study found that the prevalence of intimate partner violence, including sexual abuse, was ten times higher among cervical cancer patients compared to the general population [10]. For some women who have experienced sexual abuse, examinations and cancer-treatment procedures may feel humiliating and can provoke feelings of retraumatisation [11].

Annually, in Sweden, about 4500 women are diagnosed with cancer in the pelvic area, including cervical, endometrial, vulvar, anal, and rectal cancer [12]. Radiotherapy is a common and life-saving component of pelvic cancer treatment, essential to the continually improving cancer survival rate [12, 13]. Despite efforts to minimize side effects, cancer survivors report a high level of lifelong late adverse effects [14]. One of the most complex therapy-induced adverse late effects is sexual dysfunction [15–18], which has until now received little attention and is often underestimated by healthcare professionals [19]. Several studies have reported decreased wellbeing and distressful physical conditions involving vulvar and vaginal changes associated with nerve damage, tissue trauma and scarring [15–18]. Thus, radiotherapy induces sexual dysfunctions, and sexual abuse is an existing societal problem. However, there is limited research about the associations between radiotherapy-induced sexual dysfunctions and earlier sexual abuse among female cancer survivors. Bergmark et al. [20] previously found that, compared to healthy controls with no reported history of sexual abuse, the risk of pain during sexual activity in women who have had both cervical cancer and reported a history of sexual abuse was up to 30 times higher; their overall sexual health and wellbeing were also more negatively affected. The potential interplay of previous trauma may be reactivated by the cancer treatment experience, which rise the importance of increased knowledge in this field.

Although advances have been made in the field of sexuality research in recent decades, little is known about sexual health issues among female cancer survivors treated with pelvic radiotherapy, especially among those with a history of sexual abuse. This study had two major aims: (1) to explore sexual health aspects and wellbeing among female cancer survivors with a history of pelvic radiotherapy and (2) to determine to what extent a reported history of sexual abuse affects sexual health and wellbeing in a population-based cohort.

## Methods

### Study participants

**Fig. 1:**
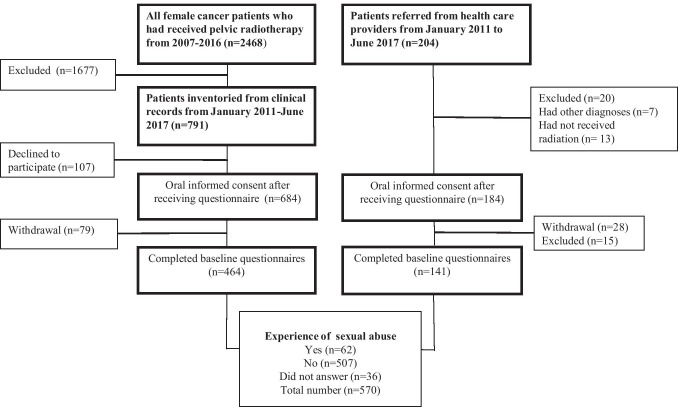
Data collection diagram including study response rate, reasons for non-participation, numbers of completed baseline questionnaires, and study participants reporting previous experience of sexual abuse

During 2011–2017, study participants were recruited from two different cohorts: (1) a population-based study cohort including all female cancer patients over 18 years treated with radiotherapy with curative intent during the years 2007–2016 at Sahlgrenska University Hospital in Gothenburg, Sweden which covers the Western Region population of 1.7 million and (2) a referred patient group including all female patients referred to the rehabilitation clinic who had been treated with curative radiotherapy (Fig. 1). Exclusion criteria were a cancer recurrence, not physically or cognitively able to understand and answer the questionnaire, and not able to understand the Swedish language. An introductory letter was sent to eligible study participants. Shortly afterwards, a research secretary phoned to give oral information and request participation.

### Study-specific questionnaire

The study-specific questionnaire, based on previously validated questionnaires, was constructed according to a clinometric methodology developed by Steineck et al. [21–23]. In short, the methodology requires a preceding quantitative phase of semi-structured interviews with persons eligible to participate in the study. A face-to-face validation had previously been carried out to ensure that the questionnaire had satisfactory internal consistency.

In total, the questionnaire consisted of 175 questions concerning demographics, wellbeing, body image, childbirth, intestinal health, urinary tract health, lymphoedema, sexual function, and sexual abuse. An explanatory text to capture various aspects and subjective experiences of sexual abuse was provided in the section containing the sexual abuse questions. Responses to the question: “Have you been subjected to any form of sexual abuse?” were dichotomised into groups: “No never” and “Yes” where the answers “Yes, I have been slightly sexually abused”, “Yes, I have been moderately sexually abused”, and “Yes, I have been severely sexually abused” were grouped as “Yes”.

Frequency, intensity, duration, and quality of each health symptom and the degree of the distress it caused were reported. Aspects of wellbeing, e.g. quality of life, feeling depressed and feeling worried or anxious were measured using a 1–7 numeric rating scale, previously demonstrated to have high co-variation and consistency with established instruments [24]. The scale had terminal descriptors of, for example, “never feeling depressed” and “always feeling depressed”. The scores were trichotomised as 1–2 “Never”, 3–5 “Sometimes”, and 6–7 “Always”. To what degree the participants were sexually active was assessed indirectly through, for example, the origin questions: “Have you noticed vaginal shortness during vaginal sex?” with optional answers ranging from “Not relevant”, “No, not at all”, “Yes, a little”, “Yes, moderate”, and “Yes, a lot”, and “Have you used lubricant during vaginal sex” with optional answers ranging from “Not relevant” to “No never”, “Yes, occasionally”, “Yes, sometimes”, and “Yes, always”. The option to fill in “Not relevant” enabled the exclusion of participants who have not had vaginal sex in the past month. Furthermore, the question “How often – approximately—did you have vaginal intercourse (or similar) on average last month?” with optional answers ranging from “Never” to “more often than 2 times per week” captures the frequency of practising vaginal sex.

The women reported the onset of menopause, use of hormone replacement therapy and use of topical oestrogen. Furthermore, aspects of sexual function such as impaired lubrication, frequency of vaginal sex, vaginal shortness and inelasticity, and superficial and deep genital pain during vaginal sex were reported. Data on cancer diagnosis and radiotherapy were collected from medical records.

### Terminology

The terms vulvar pain, genital pain, dyspareunia, and sexual pain are multifaceted constructs with diverse definitions [25, 26] and inconsistently used in the literature. Since sexual activity includes a variety of sexual practices, genital pain during sexual activity should include both vulvar (clitoral, labia, vulvar vestibule) pain and vaginal pain during penetration and/or at orgasm. In our study, we differentiated superficial and deep pain during vaginal sex and we have chosen to use the terms superficial or deep genital pain throughout this paper. When the study was conducted, we used the term vaginal intercourse or similar; however, in this article we use the term vaginal sex.

### Data analysis

The answers to the questionnaire were coded and transferred into EpiData Software version 3.1 (EpiData Association), exported to Microsoft Excel, and finally imported into the statistical program R version 3.5.2 for statistical analysis. The answers were dichotomised or trichotomised, as previously described. A subgroup of study participants with a reported history of sexual abuse was identified. In order to investigate the associations between a reported history of sexual abuse and outcomes with more than two categories, the chi-square test was applied when feasible. Otherwise, Fisher’s exact test was used. Associations between a reported history of sexual abuse and outcomes with two categories were analysed in terms of relative risks as estimated by the log-binomial model, implemented in the R function glm(). Ninety-five percent confidence intervals and the likelihood ratio test were used for inference. The statistical significance of the differences in demographical characteristics with respect to a reported history of sexual abuse was assessed using ANOVA in the cases of continuous variables and the chi-square or Fisher’s exact test was used in the cases of categorical variables. Group differences were considered significant if their associated p values were strictly less than 0.05.

### Ethical considerations

All procedures in the study involving human participants were conducted in accordance with the ethical standards of the regional ethics review board in Gothenburg (D 686–10) and with the 1964 Helsinki Declaration and its later amendments. Informed consent was obtained individually from all study participants.

## Results

### Characteristics of the study sample

In total, 570 women participated in the study. Of these, 62 (10.9%) reported a history of sexual abuse (Table 1). Women with a reported history of sexual abuse were on average younger than those with no reported history of sexual abuse (57.2 years vs. 64.9 years, p < 0.001), a higher proportion stated that they had a partner but lived alone (16.1% vs. 3.7%, p < 0.001), and they were more likely to be on sick leave (16.7% vs. 8.5%, p = 0.024). More women with than without a reported history of sexual abuse had cervical cancer, but this was not statistically significant (32.3% vs. 21.5%, p = 0.063). Of the 62 women with a reported history of sexual abuse, 33 (53.2%) ranked the degree of abuse as “mild”, 11 (17.7%) as “moderate” and 18 (29%) as “severe”. The mean age for first sexual abuse was 13.6 years with a range of 4–35 years.

**Table 1:** Demographics and clinical characteristics of female pelvic cancer survivors with and without a history of sexual abuse

Characteristics	Sexual abuse, N = 570		P-value
	No 508 (89.1)	Yes 62 (10.9)	
Age, in years			** < 0.001**
Mean (IQR^a^)	64.94 (58–74)	57.21 (51–68)	
SD^b^	12.4	12.3	
Min–max	27–94	26–76	
Marital status, N (%)			** < 0.001**
Has a partner but lives alone	19 (3.7)	10 (16.1)	
Married or living with partner	346 (68.1)	39 (62.9)	
Single	81 (15.9)	13 (21)	
Widow	62 (12.2)	0 (0)	
Employment status, N (%)			**0.024**
Disability pension	26 (5.1)	6 (10)	
Employed	137 (27.1)	21 (35)	
Housewife	4 (0.8)	0 (0)	
On sick leave	43 (8.5)	10 (16.7)	
Retired	282 (55.8)	21 (35)	
Student	3 (0.6)	1 (1.7)	
Unemployed job seeker	10 (2)	1 (1.7)	
Smoking, N (%)			0.760
Not smoking	380 (87.4)	51 (85)	
Smoking	55 (12.6)	9 (15)	
Cancer type, N (%)			0.063
Cervical cancer	109 (21.5)	20 (32.3)	
Endometrial cancer	187 (36.8)	16 (25.8)	
Vulvar cancer	19 (3.7)	1 (1.6)	
Rectal cancer	126 (24.8)	12 (19.4)	
Anal cancer	63 (12.4)	11 (17.7)	
Other	4 (0.8)	2 (3.2)	
Cancer treatment, N (%)			0.955
External radiotherapy with and without brachytherapy	133 (88.7)	17 (11.3)	
Surgery with external radiotherapy with and without brachytherapy	375 (89.3)	45 (10.7)	
Degree of sexual abuse^c^, N (%)			
A little		33 (53.2)	
Moderate		11 (17.7)	
Severe		18 (29)	
Repeated sexual abuse, N (%)			
No		44 (71)	
Yes		17 (27.4)	
Not relevant		1 (1.6)	
Incest, N (%)			
No		56 (90.3)	
Yes		6 (9.7)	
Age at first sexual abuse (IQR^a^)			
Mean		13.6 (8.2–17)	
SD		6.6	
Min–max		4–35	
Exposure to sexual abuse has affected sexual life, N (%)			
Moderate or a lot		20 (32.3)	
Not at all or a little		34 (54.8)	
Not relevant		5 (8.1)	

*N*, numbers

^a^*IQR*, interquartile range

^b^*SD*, standard deviation

^c^Experience of sexual abuse subjectively assessed by the study participants with the range: a little, moderate, and severe

Note: Internal dropouts range: 0–14.55%

*P*‐values in bold font indicate statistical significance (≤0.05)

### Aspects of wellbeing and self-esteem

A statistically significantly higher proportion of women with than without reported a history of sexual abuse reported “always feeling depressed” (19.4% vs. 9%, p = 0.007) and “always feeling worried or anxious” (22.6% vs. 11.8%, p = 0.007) in the past 6 months (Table 2). About 70% of all women reported decreased self-esteem. A greater proportion of women with than without a reported history of sexual abuse reported increased self-esteem after cancer treatment (28% vs. 15.9%, p = 0.009).

**Table 2:** Self-reported aspects of wellbeing and self-esteem in female cancer survivors with and without experience of sexual abuse

Aspects assessed	Experience of sexual abuse N (%)		P-value
	No N = 499	Yes N = 62	
Level of QoL^a^, N (%)			0.134
No QoL at all or very low	46 (9.2)	7 (11.3)	
Moderate	303 (60.8)	44 (71)	
Very high	149 (29.9)	11 (17.7)	
How often feeling depressed^b^, N (%)			**0.007**
Always	45 (9)	12 (19.4)	
Sometimes	243 (48.7)	34 (54.8)	
Never	211 (42.3)	16 (25.8)	
How often feeling worried or anxious^b^, N (%)			**0.007**
Always	59 (11.8)	14 (22.6)	
Sometimes	240 (48.2)	34 (54.8)	
Never	199 (40)	14 (22.6)	
Self-esteem^a^, N (%)			0.285
Low	33 (6.9)	6 (9.7)	
Moderate	294 (61.3)	42 (67.7)	
High	153 (31.9)	14 (22.6)	
Change in self-esteem after cancer and cancer treatment, N (%)			**0.009**
Not relevant	39 (13.5)	2 (4)	
Yes, decreased	204 (70.6)	34 (68)	
Yes, increased	46 (15.9)	14 (28)	

*N*, numbers; *QoL*, quality of life

^a^Patient-reported answers with the range 1–7 and classified as follows: 1–2 “Low”, 3–5 “Moderate”, and 6–7 “High”

^b^Patient-reported answers with a range 1–7 classified as follows: 1–2 “Never”, 3–5 “Sometimes”, and 6–7 “Always”

Note: Internal dropouts range: 0–6.61%

*P*‐values in bold font indicate statistical significance (≤0.05)

### Sexual health aspects

More women with than without a reported history of sexual abuse used topical oestrogen (34.5% vs. 21.1%, p = 0.100) and had noticed “moderate or a lot” of vaginal shortness during vaginal sex (51.6% vs. 34.6%, p = 0.073, RR 1.49, CI 0.96–2.14), although the differences were not statistically significant (Table 3).

**Table 3:** Self-reported sexual health aspects in women with and without a reported history of sexual abuse

Aspects assessed	Experience of sexual abuse, N (%)		P-value	RR (CI)
	No	Yes		
Onset of natural menopause before cancer treatment^a^, N (%)			**0.004**	
No	104 (21)	23 (38.3)		
Yes	391 (79)	37 (61.7)		
Use of hormone replacement (oestrogen^a^), N (%)			0.100	
No	342 (70.1)	32 (58.2)		
Yes, systemic hormone therapy	40 (8.2)	4 (7.3)		
Yes, topical oestrogen	106 (21.7)	19 (34.5)		
Feeling of sexually attractiveness^a^, N (%)			0.720	1.04 (0.90–1.15)
Not at all or a little	383 (80.5)	50 (83.3)		
Moderate or a lot	93 (19.5)	10 (16.7)		
Satisfied with partner as a friend/fellow human being^b^, N (%)			**0.010**	3.45 (1.37–7.86)
Not at all or a little	14 (4.2)	7 (14.6)		
Moderate or a lot	317 (95.8)	41 (85.4)		
Satisfied with partner as a lover^b^, N (%)			0.769	1.12 (0.35–2.64)
Not at all or a little	27 (11.2)	4 (12.5)		
Moderate or a lot	215 (88.8)	28 (87.5)		
Noticed vaginal shortness during vaginal sex^b^, N (%)			0.073	1.49 (0.96–2.14)
Moderate or a lot	65 (34.6)	16 (51.6)		
Not at all or a little	123 (65.4)	15 (48.4)		
Noticed vaginal inelasticity during vaginal sex^b^, N (%)			0.282	1.28 (0.87–1.27)
Moderate or a lot	65 (35.5)	15 (45.5)		
Not at all or a little	118 (64.5)	18 (54.5)		
Use of lubricant during vaginal sex^b^, N (%)			**0.025**	1.54 (1.10–1.99)
Not at all or occasionally	113 (55.1)	9 (31)		
Sometimes or always	92 (44.9)	20 (69)		
Superficial genital pain during vaginal sex^b^, N (%)			0.614	1.2 0.70–1.82
Moderate or a lot	62 (35.8)	12 (42.9)		
Not at all or a little	111 (64.2)	16 (57.1)		
Deep genital pain during vaginal sex^b^, N (%)			**0.011**	**2.07** (1.24–3.16)
Moderate or a lot	42 (25.1)	13 (52)		
Not at all or a little	125 (74.9)	12 (48)		
Level of distress if genital and sexual pain during vaginal sex persists^b^, N (%)			0.205	1.03 (0.97–1.23)
Moderate or a lot	134 (85.4)	28 (93.3)		
Not at all or a little	23 (14.6)	2 (6.9)		
How often vaginal sex^a^, N (%)			0.246	0.6 (0.29–1.32)
Never, a few times or less than 1–2 times a month	409 (89.7)	52 (83.9)		
About 1–2 times a week	47 (10.3)	10 (16.1)		
Level of distress if current problems with vaginal sex persist^b^, N (%)			0.418	1.1 (0.90–1.29)
Moderate or a lot	160 (72.4)	32 (80)		
Not at all or a little	61 (27.6)	8 (20)		
Overall satisfaction with sexual life^b^, N (%)			0.612	1.08 (0.85–1.31)
Moderate or a lot	93 (37.1)	15 (31.9)		
Not at all or a little	158 (62.9)	32 (68.1)		
Level of distress if overall problems with sexual life persist^b^, N (%)			0.881	1.04 (0.84–1.23)
Moderate or a lot	173 (71.5)	35 (74.5)		
Not at all or a little	69 (28.5)	12 (25.5)		

*N*, numbers; *RR*, relative risk; 95% confidence interval (CI). Patient-reported answers were dichotomised and merged as follows: “Not at all; Yes, a little” as indicating not at all or a little and “Moderate; A lot” as indicating moderate or a lot

^a^Includes the total sample

^b^Women who reported “Not relevant” were excluded from the analysis

Note: Internal dropouts range: 23.48–24.9%

*P*‐values in bold font indicate statistical significance (≤0.05)

Twice as many women with than without a reported history of sexual abuse reported deep genital pain during vaginal sex (52% vs. 25.1%, p = 0.011, RR 2.07, CI 1.24–3.16). Almost all women reported “moderate or a lot” of distress with persistent genital pain during vaginal sex (93.3% vs. 85.4%, p = 0.379).

### Genital pain and associations with physical aspects and wellbeing

Superficial genital pain was more common among rectal cancer survivors (27%) than among women with other diagnoses, while deep genital pain was more common among cervical cancer survivors (41.8%) (Table 4). A statistically significantly higher proportion of women with than without vaginal inelasticity reported both superficial genital pain (68.1% vs. 31.8%, p = 0.001) and deep genital pain (66.6% vs. 33.3%, p < 0.001).

**Table 4:** Bivariable analysis of possible predictors for genital pain during vaginal sex among women treated with pelvic radiotherapy

	Superficial genital pain^a^, N = 205 (%)		P-value	Deep genital pain^a^, N = 226 (%)		P-value
	Yes n = 74 (36.6)	No n = 128 (63.4)		Yes n = 55 (28.6)	No n = 137 (71.4)	
Diagnosis			0.122			**0.016**
Anal cancer	16 (21.6)	14 (10.9)		9 (16.4)	21 (15.3)	
Cervical cancer	18 (24.3)	34 (26.6)		23 (41.8)	28 (20.4)	
Endometrial cancer	17 (23)	46 (35.9)		9 (16.4)	47 (34.3)	
Rectal cancer	20 (27)	31 (24.2)		12 (21.8)	37 (27)	
Vulvar cancer	3 (4.1)	2 (1.6)		2 (3.6)	2 (1.5)	
Other	0 (0)	1 (0.8)		0 (0)	2 (1.5)	
Topical oestrogen use			0.101			0.071
Yes	28/74 (37.8)	33/128 (25.7)		37(38.6)	17 (61.4)	
No	46/74 (62.1)	95/128 (74.2)		35 (24.4)	102 (75.6)	
Vaginal shortness			**0.031**			** < 0.001**
Yes	31/66 (47.7)	34/114 (29.8)		37/54 (68.5)	29/127 (22.8)	
No	34/66 (51.5)	80/114(70.2)		17/54 (31.4)	103/124 (83)	
Vaginal inelasticity			** < 0.001**			** < 0.001**
Yes	45/66 (68.1)	22/118 (18.6)		36/54 (66.6)	26/129 (20.2)	
No	21/66 (31.8)	96/118 (81.3)		18/54 (33.3)	103/129 (79.9)	

N (number) and proportion (%) of women are presented and include only women who had practised vaginal sex. P-values in bold print

^a^Patient-reported answers were dichotomised as follows: “No, not at all; Yes, a little” as indicating no and “Yes, moderate; Yes, a lot” as indicating yes

Note: Internal dropouts range: 23.48–24.9%. Includes only women who have reported sexual activity

## Discussion

Our results indicate that more women with than without a history of sexual abuse reported impaired sexual health and wellbeing. Deep genital pain during vaginal sex was one of the sexual health aspects that were statistically significantly higher among women with a reported history of sexual abuse compared to women without a reported history of sexual abuse. We found that more women with than without a history of sexual abuse reported having felt depressed or anxious. Substantially, our results are consistent with the study by Bergmark et al. [20], which shows a statistically significant higher risk and a synergistic effect for genital pain during vaginal sex among cervical cancer survivors with a history of both pelvic radiotherapy and sexual abuse compared to healthy controls. Our study adds to previous research that shows that women treated with radiotherapy experience long-term and distressing sexual dysfunction and decreased wellbeing, regardless of the origin of the pelvic cancer.

As expected, the mean age was lower among women with than without a reported history of sexual abuse. HPV infections are extremely common in young women during the first decade of their sexual relations (9), although other co-factors, such as immunological aspects, are considered necessary for the development of cancer [8]. It is estimated that half of high-risk HPV infections that develop into cancer are acquired by age 21 years, 75% by age 31 years and more than 85% by age 40 years [27]. Persistent infections and pre-cancer establish within 5–10 years while invasive cancer arises over many years [9]. An HPV-induced cancer relates to sexual abuse only in some cases [28]. Since one of the coping strategies for dealing with sexual abuse trauma is to avoid regular cervical screening [29], early detection and treatment is not possible which may have negative consequences later [30]. Victims of sexual abuse may experience cancer treatment procedures as invasive and humiliating, which can trigger thoughts and emotions associated with the original abuse. This may lead to further unhealthy outcomes due to negative effects on mental and physical health [20, 29], and retraumatisation [11].

For all women in the study cohort, vaginal shortness was associated with genital pain, previously explained by Hofsjö et al. [18] as being caused by radiotherapy-induced fibrosis. Vaginal shortness can be induced not only by radiotherapy, but also by surgery and resection of the vagina. However, we found that genital pain during sexual activity was less likely among women who did not use topical oestrogen, which is contrary to our clinical experience that topical oestrogen is helpful in reducing discomfort and genital pain. However, we have no information concerning what motivates topical oestrogen use and speculate that this finding may reflect women not having received adequate information [31]. Lubricant use during vaginal sex was more common among women with a reported history of sexual abuse, which may reflect that they have developed a higher sexual awareness motivating actions to prevent pain. We suggest individualized counselling and holistic management to facilitate adherence to self-care recommendations such as use of topical oestrogen, lubricant and a vaginal dilator.

Contrary to our preconceptions and clinical experience, women treated for rectal cancer had a high prevalence of superficial genital pain; one could instead expect high prevalence of deep genital pain during sexual activity associated with the anatomical changes caused by surgery. In one study, having a permanent stoma after colorectal cancer was found to be associated with genital pain during vaginal sex [32], findings probably related to a retrograde vaginal direction following rectal amputation that makes vaginal sex complicated for some women. In line with previous research [32, 33], we found that pain during vaginal sex was less likely among women who had not noticed vaginal shortness or inelasticity. It is not only direct scarring and loss of elasticity and blood supply to the genitals that radiotherapy induces; abrupt premature ovarian failure due to oophorectomy or pelvic radiotherapy, and exacerbation of normal menopause during and after radiotherapy also lead to severe oestrogen deprivation and genitourinary atrophy [15, 18, 34].

The manifestation of genital pain after pelvic radiotherapy are similar to those of genital pain among women in the general population, including provoked vestibulodynia, pelvic floor dysfunction, vulvar dermatoses and the genitourinary syndrome of menopause. These complex conditions are understood in the context of female sexual response, meaning that disruption at any point of the response cycle can contribute to, cause and maintain genital pain [3, 35]. Irrespective of where the pain originates from, the therapy is similar and includes recommendations such as oestrogen use, dilator therapy, relaxation treatment of pelvic floor muscles, open communication in the relationship, and more educational interventions to understand the changes of the anatomy and physiology, and support exploration of sexual practices [31, 36].

The overall low wellbeing reported among all female cancer survivors agrees with previous studies of cervical [17], endometrial, vulvar, vaginal, rectal, and anal cancers [37]. The majority in our study reported decreased self-esteem after cancer treatment, also previously reported by half of all women in a study concerning early cervical cancer [17]. While decreased self-esteem would be expected, increased self-esteem was reported by one-third of the women with a reported history of sexual abuse. Positive changes may follow survival of a life-threatening disease. As found in a previous study and from our clinical experience, some patients seem to take something positive from the cancer experience, which might increase feelings of strength and confidence [38]. The high proportion of internal dropout in the self-esteem question in our study impeded further analysis, which in our consideration is needed to understand changes in self-esteem, both increased and decreased.

In the past decades, attitudes related to sexuality have changed in western countries leading to less taboo and stigma. Despite this, screening for sexual health and sexual abuse is still not routine in oncology settings. Previous research reveals that cancer-related sexual problems often remain unidentified despite patients’ wishes for help [34, 39], which implies the need for further research and clinical intervention. Our study indicates that sexual abuse may predict impaired sexual health and wellbeing in female cancer survivors, emphasising the need for supportive care of these women. Although sexuality is considered as one of the high-incidence areas of nursing care [40], lack of professional confidence in dealing with these issues, and a complex healthcare system, impedes optimal sexual healthcare. In our opinion, the considerable magnitude of the problem stresses the importance of necessary resources and skills, including continuing education programs for healthcare professionals. Furthermore, other radiotherapy-induced diseases and conditions, such as impaired intestinal and urinary tract health, may negatively affect sexual health among female pelvic cancer survivors and are aspects that need to be considered.

### Limitations and strengths

The subgroup in our study was relatively small but reflects the proportion of women with a reported history of sexual abuse in the general European population [5] to which this study may be generalized. However, the proportion is way below that reported in the WHO global report [4], which includes low-income countries where women, in general, are more exposed to intimate partner violence [29]. Since advances in radiotherapy techniques mean that the outcomes of women treated in the early 2000s may differ from those treated in 2016, the wide range of time in our study since completion of radiotherapy, 6 months to 10 years, may be a weakness. On the other hand, this has contributed to an understanding of the progressive and long-term effects of pelvic radiotherapy and health consequences.

The internal dropouts may affect the consistency, since the reasons for participants not responding are unknown. We believe that some women might perceive the questions as intrusive, and factors such as age, culture and norms may interplay. Furthermore, our study would benefit from questions concerning sexual practices other than vaginal sex to include the wider range and perspective of female sexuality. Memory-induced problems represent a threat to the internal validity and, for some study participants, if only a short time has passed since completion of radiotherapy their answers may be influenced by symptoms from acute side effects.

To our knowledge, this is the first study addressing sexual health problems in female pelvic cancer survivors treated with radiotherapy regardless of specific cancer diagnosis. No previous study has determined the extent to which the history of sexual abuse affects cancer survivors treated for pelvic radiotherapy. The methodological strengths include the large population-based study cohort and high participation rate. In addition, the possibility of answering the self-reported questionnaire anonymously and in private reduces the risk of potential therapist-induced bias. Self-reported data is known to provide a wider range of responses than data collected using other instruments [41]. The clinometric method described in this and other research projects [14, 15, 21, 23] provides a high validity.

The observational study design enabled an exploration of the total population-based cohort and the performance of subgroup analysis. To handle bias and confounding, we employed a hierarchical step-model, developed for the utilisation of epidemiological methods in the cancer survivorship field [22]. We are aware that not only radiotherapy but also hysterectomy and rectal surgery may cause vaginal shortness and inelasticity [42, 43] and approximately 74% of the study participants had undergone surgery as part of their treatment. Furthermore, our study was developed and carried out in Sweden, which may limit the generalisability to low-income countries due to a lack of screening programs for HPV in underdeveloped healthcare systems. The results are probably applicable to populations in high-income countries.

### Conclusions

This study highlights that sexual abuse has a negative impact on sexual health outcomes and other health aspects, and that affected women need support in their cancer treatment process. If unhealthy outcomes of cancer treatment procedures are to be prevented, our results may be worth to consider. We suggest routine screening in oncology settings to identify women with a history of sexual abuse before, during, and after cancer treatment. This requires wider multidisciplinary teamwork in which oncology nurses play a pivotal role. Hopefully, this can improve supportive care and prevent retraumatisation among affected women. More studies are needed to explore the impact on sexual health of other treatment-induced side effects, such as intestinal and urinary tract symptoms, lymphoedema and pain. Furthermore, it is important to take action to promote patients’ improved sexual health and wellbeing or refer them to experts in the field such as psychotherapists, sexual health counsellors, sexologists, pelvic floor physiotherapists, and peer-support.

## Data Availability

Data are available on reasonable request.

## Code availability

The datasets generated and analysed during the current study are available from the corresponding author on reasonable request.
